# Contingency management to promote treatment engagement in comorbid alcohol use disorder and alcohol‐related liver disease: Findings from a pilot randomized controlled trial

**DOI:** 10.1111/acer.70018

**Published:** 2025-03-10

**Authors:** Sofia Hemrage, Nicola Kalk, Naina Shah, Stephen Parkin, Paolo Deluca, Colin Drummond

**Affiliations:** ^1^ Department of Addictions, Institute of Psychiatry, Psychology and Neuroscience King's College London London UK; ^2^ South London and Maudsley NHS Foundation Trust London UK; ^3^ Institute of Liver Studies King's College Hospital London UK; ^4^ Department of Public Health, Environments and Society London School of Hygiene and Tropical Medicine London UK

**Keywords:** alcohol use disorder, alcohol‐related liver disease, contingency management, feasibility, treatment engagement

## Abstract

**Background:**

Alcohol‐related liver disease (ARLD) is a leading cause of preventable death and health inequalities. Evidence‐based interventions for comorbid alcohol use disorder (AUD) and ARLD remain limited, and only a small proportion of this clinical population engages with treatment. There is a need to improve patient outcomes by bridging this gap through novel, person‐centred interventions. Contingency management (CM) is a psychosocial intervention that involves gradual, increasing incentives upon the completion of treatment‐related goals, such as treatment attendance. This single‐centre, randomized pilot trial of voucher‐based CM was conducted to promote treatment engagement in comorbid AUD and ARLD.

**Methods:**

Thirty service users were recruited from an inpatient setting, offered integrated liver care (ILC) and allocated to ILC only or ILC + CM. Primary outcomes included feasibility criteria (recruitment, study retention post‐randomization, completeness of data and protocol fidelity). Secondary outcome data on engagement, alcohol intake, and liver function were also collected. Data were gathered at baseline, post‐ILC, and 12 weeks post‐ILC and analyzed through descriptive statistics.

**Results:**

The feasibility of the research was subject to challenges inherent to conducting applied health research in a real‐world clinical setting. The recruitment and retention rates were 73.20% and 36.70%, respectively. All participants received CM per protocol. An increasing trend in engagement was observed in the ILC + CM compared to ILC only (67% vs. 33%). A trending 76% reduction in alcohol intake and an overall improvement in liver outcomes were observed among participants engaging with the trial, with no significant differences between control and treatment groups.

**Conclusion:**

Overall, the CM intervention was feasible to deliver and appears promising in improving outcomes in individuals with comorbid AUD and ARLD. Aspects related to recruitment, study retention post‐randomization, and protocol fidelity need to be further adapted before proceeding with a definitive trial.

## Graphical abstract

This pilot trial explored the feasibility of contingency management to promote treatment engagement in patients with comorbid alcohol use disorder and alcohol‐related liver disease. Participants were offered integrated care, and those in the intervention arm were additionally offered a voucher‐based contingency management intervention. An increasing trend in engagement was observed in the intervention group. A reduction in alcohol intake and an overall improvement in liver outcomes were observed among participants engaging with the trial.

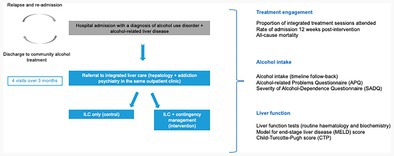

## INTRODUCTION

Alcohol‐related liver disease (ARLD) refers to a broad spectrum of clinical presentations and histopathological lesions resulting from prolonged, harmful alcohol intake, as well as an interplay between demographic, genetic, and environmental risk factors. This spectrum encompasses a range of diagnoses, including steatosis, fibrosis, cirrhosis (with or without decompensation), hepatitis, and acute‐on‐chronic liver failure (Hernández‐Évole et al., 2024).

The overall global prevalence of ARLD is 4.80%, with variations across regions (Niu et al., 2023). Age‐standardized death rates for ARLD are 4.50 per 100,000 (95% CI 3.80, 5.30) (Abbafati et al., 2020). ARLD mortality was associated with patterns of alcohol intake, being higher in central Europe (42%) compared to the Eastern Mediterranean region (8%). These figures might, however, be an underestimation of the global burden of ARLD due to differences in registration systems, access to health services, and diagnostic tools (Huang et al., 2023).

Between 2009 and 2015, the prevalence of ARLD in the United States of America increased by 43% (Niu et al., 2023). ARLD mortality rates have particularly increased among younger age groups (25–34 years) and females (Ilyas et al., 2023). Local and national data from the Office for Health Improvements & Disparities (England) reported a surge in ARLD admissions and mortality, aggravated by the onset of COVID‐19 (Office for Health Improvement and Disparities, 2024). In 2022, 76% of alcohol‐specific deaths in England were attributed to ARLD (Office for National Statistics, 2024).

For patients with comorbid AUD and ARLD, engagement with alcohol treatment can improve outcomes such as liver function, health‐related quality of life, hospitalization rates, and mortality (Rogal et al., 2020). However, this high‐risk clinical population has suboptimal engagement with treatment. After receiving an ARLD diagnosis, only 10%–15% of patients engage with treatment services (Mellinger et al., 2019; Rogal et al., 2020). Recent research noted that past 12‐month treatment utilization was more likely among patients of younger age, with current clinically significant anxiety or depression (Luk et al., 2025). More specifically, a retrospective cohort study found that, in the previous 12 months, 11% of patients with ARLD accessed behavioral support, 2% were prescribed pharmacotherapeutic treatment, and only 1% received integrated pharmacological and psychosocial management (Alexandre et al., 2023). Non‐engagement was also found to be associated with ongoing alcohol use, hepatic decompensation, and incidence of hepatocellular carcinoma. As a result of the chronic nature of AUD and the dose–response relationship between alcohol intake and liver injury, sustained engagement with alcohol treatment is central to improving health‐related quality of life in this clinical population.

### Psychosocial interventions in comorbid AUD and ARLD: contingency management

Interventions targeting engagement and abstinence outcomes may provide an opportunity for improving patient outcomes and health‐related quality of life in comorbid AUD and ARLD. A systematic review comprised of 13 studies with 1945 patients with ARLD evaluated the efficacy of psychosocial interventions to induce and maintain abstinence in comorbid AUD and ARLD (Khan et al., 2016). The included studies trialed combined psychotherapy with cognitive behavioral therapy (CBT), Motivational Enhancement Therapy (MET), motivational interviewing, supportive therapy, and psychoeducation alone or in combination with treatment as usual. The review found that psychotherapy combined with CBT, MET, and integrated care with CBT induced abstinence; no psychosocial intervention was effective in maintaining abstinence. However, returning to drinking was lower with integrated care. The findings noted that integrated, multidisciplinary approaches appear promising in addressing this clinical population's unique needs and preferences. This research also pointed to the need to trial novel, tailored psychosocial interventions to improve patient outcomes in comorbid AUD and ARLD.

Contingency management (CM) is a psychosocial intervention that can support positive behavior change, with its principles founded upon the underpinnings of behaviorism and operant conditioning. By employing positive reinforcement, CM is based on the notion that a desired behavior reinforced in close temporal proximity to its occurrence will increase in frequency (Petry, 2011). This can increase the frequency of voluntary, health‐promoting behaviors such as abstinence, reduced intake, or treatment engagement. A growing body of empirical evidence has supported the effectiveness of CM across a range of target behaviors (substance or non‐substance‐focused), clinical populations receiving substance use treatment (cocaine, opiates, polysubstance use, cannabis, alcohol or nicotine), modes of incentive (clinical privileges, prizes, vouchers) and schedules (Lussier et al., 2006; Pfund et al., 2024).

Psychosocial interventions such as CM can address barriers associated with pharmacological approaches and low treatment engagement. Accordingly, a multi‐centre, three‐arm parallel‐group randomized controlled trial (RCT) has supported the scope of CM to support medication adherence (Donoghue et al., 2023). However, no previous research has evaluated a CM intervention among a clinical population with comorbid AUD and ARLD, whose effective management often involves complex, multidisciplinary treatment regimens (Khan et al., 2016).

### Evidence gap and current study

While CM has been well established in substance use treatment, limited research has been conducted in AUD and clinical settings outside of the USA (Cohen et al., 1971; Petry et al., 2000). In addition, no previous research has evaluated a CM intervention among a clinical population with comorbid AUD and ARLD, whose effective management often involves complex, multidisciplinary treatment regimens (Hemrage et al., 2023; Khan et al., 2016). This gap reflects a need for novel interventions simultaneously targeting AUD and ARLD outcomes to be trialed in a real‐world context (Lee et al., 2024; Mellinger et al., 2021; Singal et al., 2022).

To address this gap, a pilot trial was conducted to explore the feasibility of delivering CM to promote engagement with multidisciplinary treatment. The primary aim was to characterize the demographic profile of the clinical population of interest and generate data on feasibility to inform progression to a definitive RCT. The pilot trial also aimed to generate exploratory data on treatment engagement, alcohol use, and liver function following a CM intervention.

## METHODS

### Design and procedures

**FIGURE 1:**
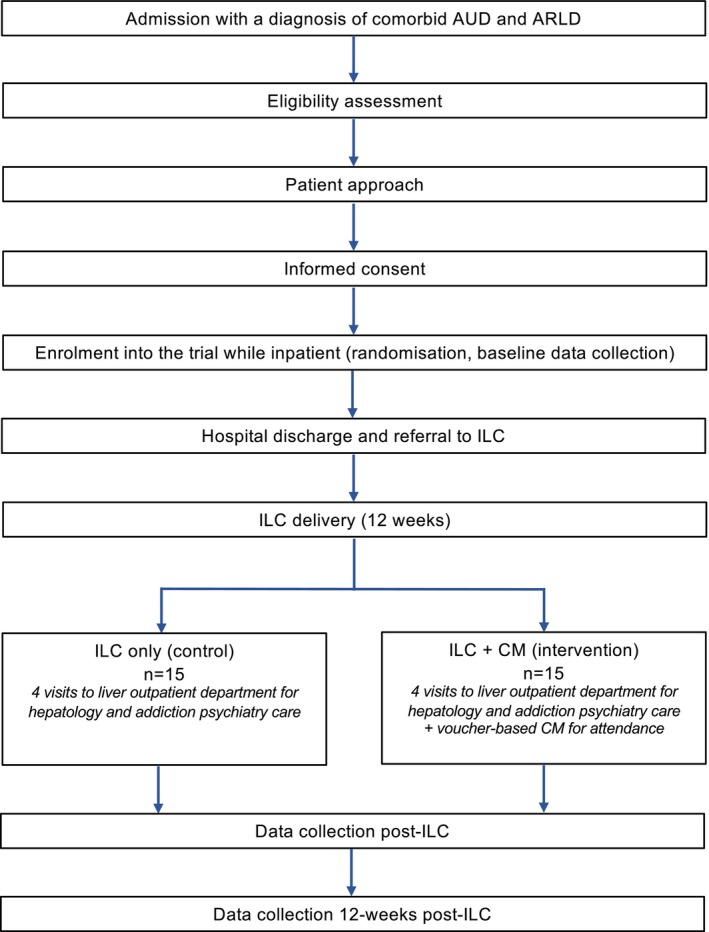
Study flow diagram. ARLD, alcohol‐related liver disease; AUD, alcohol use disorder; CM, contingency management; ILC, integrated liver care.

A single‐centre, randomized pilot trial of Integrated Liver Care (ILC) only (control) versus ILC + CM (intervention) was conducted at King's College Hospital NHS Foundation Trust (KCH) in partnership with the Alcohol Care Team (ACT) and Liver Outpatient Department (LOPD). The study aimed to explore the acceptability and feasibility of delivering CM to promote treatment engagement among the target clinical population. The present article reports on feasibility data (quantitative) and a study flow diagram can be found in Figure 1.

Qualitative data generated on acceptability are reported in a separate article (Hemrage et al., 2024c). Through a theory‐informed approach, the qualitative component of the trial demonstrated positive views toward CM (intervention coherence, ethicality, self‐efficacy, perceived effectiveness and affective attitudes), with its core components matching participants' preferences and needs. CM was considered an appropriate and person‐centred intervention for this clinical population.

The pilot trial has been peer‐reviewed following the regulatory and governance requirements set by King's College London and King's College Hospital Research and Innovation (R&I). A favorable opinion was granted by Camden and Kings Cross NHS Research Ethics Committee (reference 22/LO/0744). The trial has been registered on the Open Science Framework (identifier DOI 10.17605/OSF.IO/AVKYJ) and ClinicalTrials.gov database (identifier: NCT06183710) (ClinicalTrials.gov, 2023; Hemrage, 2022). As part of the protocol, a record of serious adverse events (SAEs) was kept and reported to the sponsor. The CONSORT checklist for the trial is provided as Appendix S1.

The trial was conducted under the principles of the Declaration of Helsinki, Good Clinical Research Practice (GCP) and General Data Protection Regulation (GDPR) and additional relevant regulatory requirements (UK Government, 2018; World Health Organization, 2005; World Medical Association, 2013). Written informed consent was sought before any data was collected. To compensate participants for their time, a £15 *honorarium* was offered both at baseline and post‐ILC data collection points.

### Study population, sampling and randomization

Consecutive sampling was used to recruit 30 adult participants presenting a comorbid diagnosis of AUD and ARLD from an inpatient setting, admitted with an acute liver episode and not referred for liver transplantation at the time of recruitment. A sample size of 30 participants was decided upon after reviewing available guidance for feasibility and pilot studies (Eldridge et al., 2016). Recruitment was set for an initial period of 12 weeks. Due to suboptimal recruitment rates, additional sites were added (the emergency department at KCH and Princess Royal University Hospital, King's College Hospital NHS Foundation Trust), and the recruitment period was extended until the target sample size was achieved (35 additional weeks).

The following inclusion criteria were set:

- Aged 18 or above.
- Diagnosis of AUD was obtained from electronic health records and further suggested by an AUDIT score above 19 (Saunders et al., 1993).
- Diagnosis of ARLD (non‐severe and severe alcohol‐related hepatitis, cirrhosis or decompensated liver disease), validated through clinical examination or laboratory tests including FibroScan and/or radiological investigations.
- Presentation to the emergency department or admission to the inpatient setting.
- Referral to ILC upon discharge.
- Willingness and capacity to provide informed consent to take part in the trial.
- Ability to communicate in English independently.

Cognitive impairment, as evidenced by the inability to provide voluntary informed consent, was an exclusion criterion. Patients who were dependent upon substances other than alcohol, tobacco, or cannabis were not eligible. Pregnancy also precluded participation in the pilot trial.

Participants were randomized following a string randomization approach (1:1 ratio) using a *Stata* code developed by the study statistician (StataCorp, 2021). The allocation sequence was printed on separate pages and sealed into individual, numbered (1–30) envelopes. The allocation was concealed until the entry of a new participant. After enrollment, the researcher (SH) opened an envelope corresponding to the participant's assigned number. After this stage, double blinding was not possible due to the design of the study. Given the nature of the CM intervention, the participants, the ILC consultants (NJK, NS), and the researcher (SH) conducting the assessments were not blinded. The remaining members of the research team (SP, PD, CD) were blinded to treatment allocation.

### Interventions

All participants were offered four ILC appointments at the LOPD over 12 weeks. These involved the provision of hepatology and addictions psychiatry within the same facility. Multidisciplinary treatment and co‐location of healthcare providers have been noted as optimal in managing ARLD (López‐Pelayo et al., 2019). Hepatology care entailed the delivery of regular liver health education, management of decompensation‐related symptoms, referral to dietetics services, and elective paracentesis, provided by the ILC hepatology consultant. Psychiatric care comprised motivational interviewing, relapse prevention techniques, referral to support services, and prescription of anti‐craving pharmacotherapy. These were provided by the ILC addictions psychiatry consultant. The consultants are part of the ACT and met daily for clinical supervision to discuss clinical cases and ensure treatment fidelity.

The employed CM intervention has been described in detail in a previous publication and is summarized in Table 1 (Hemrage et al., 2024c). The CM intervention aimed to promote treatment attendance and was delivered by the ILC consultants during appointments in the clinic. The incentives consisted of multi‐outlet vouchers exchangeable for material goods. Participants in the intervention group could receive a maximum of £120 if attending all four ILC visits, with the total amount corresponding to the number of completed sessions. For each visit, a £15 was given, with gradually increasing bonuses (£10, £20, £30) for attending at least two ILC visits (the benchmark for engagement defined in the protocol). Engagement with treatment was measured by confirming attendance at the four sessions in the participants' respective electronic health records. Upon completion of the study, the incentives were discontinued, but participants were offered further ILC on a case‐by‐case basis.

**TABLE 1:** Characteristics of the voucher‐based CM intervention adopted in the pilot trial.

CM feature	Intervention characteristics
Target behavior	Engagement with ILC, measured via attendance
Target population	Service users with comorbid AUD and ARLD
Choice of incentive	Multi‐outlet vouchers
Magnitude of incentive	Maximum of £120
Duration of incentive	12 weeks
Frequency of delivery	4 times during ILC (12 weeks), delivered in person by ILC consultants
Timing of delivery	Delivered during ILC

The CM intervention was informed by a previous multicenter, three‐arm, parallel‐group, randomized controlled trial assessing the effectiveness of adjunctive medication management and CM to enhance adherence to acamprosate for alcohol dependence (Donoghue et al., 2023). The adaptation of the CM intervention was informed through the available literature, consultation with healthcare providers, and members of the public from the earliest stages of research planning.

### Data collection and outcomes

#### Feasibility outcomes

Feasibility outcomes were collected to inform the progression to the next stage of research (effectiveness and cost‐effectiveness). These included data on recruitment, study retention post‐randomization, completeness of data, and protocol fidelity:

- *Recruitment:* eligibility rate and recruitment rate;
- *Study retention post‐randomization:* retention rate (attending at least 50% of scheduled study visits);
- *Completeness of data:* proportion of planned assessments completed at each time point (baseline, post‐ILC, 12 weeks post‐ILC);
- *Protocol fidelity:* time, in days, between the date of discharge and the first study visit (14 ± 7 working days per protocol), and duration, in weeks, of intervention delivery (12 weeks/84 ± 28 working days per protocol).

Each of these criteria was assessed according to a “traffic light system” proposed by Avery et al. (2017). The framework proposes three classifications (red, when there are intractable aspects that cannot be addressed; amber; when there are remediable aspects that need to be addressed before proceeding; green no concerning aspects that impact trial performance) that can inform the viability and scalability of the research.

#### Exploratory outcomes

The primary focus of the pilot trial was to inform the feasibility criteria outlined above. For future definitive research, the exploratory outcomes were related to the efficacy of CM to improve treatment engagement, alcohol use, and liver function.

Routine hematology and biochemistry were extracted from electronic health records (where available). Laboratory testing was not implemented as part of the protocol; this was aimed at minimizing research burden and to explore the availability of the data in routine practice. A data collection schedule for the outcomes listed above is provided in Table 2.

**TABLE 2:** Data collection schedule.

Measurement	Baseline	Visit 1	Visit 2	Visit 3	Visit 4	12 Weeks post‐ILC
Time after discharge		2 weeks (±7 days)	4 weeks (±7 days)	8 weeks (±14 days)	12 weeks (±14 days)	24 weeks (±28 days)
Engagement^a^		×	×	×	×	
Alcohol‐related admissions		×	×	×	×	×
All‐cause mortality rate		×	×	×	×	×
TLFB	×				×	×
Percentage days of abstinent					×	×
Rate of return to any drinking					×	×
Abstinence rate					×	×
APQ	×				×	×
SADQ	×				×	×
Liver function and biomarkers^b^	×				×	×
MELD 3.0 score^c^	×				×	×
CPG score^c^	×				×	×

Abbreviations: APQ, Alcohol Problems Questionnaire; CPG score, Child‐Pugh score; ILC, integrated liver care; MELD score, Model for End‐Stage Liver Disease 3.0 score; SADQ, Severity of Alcohol Dependence Questionnaire; TLFB, timeline followback.

^a^ Data extracted from electronic health records.

^b^ Routine biochemistry and hematology data extracted from electronic health records, when available.

^c^ Calculated for participants presenting cirrhosis and decompensated liver disease.

Where possible, data were collected at baseline, post‐ILC (visit 4) and 12 weeks post‐ILC. Proposed patient outcomes included:

- Engagement (attendance): proportion of patients engaging with ILC post‐randomization, per arm, as reported in electronic health records. Per protocol, the benchmark for engagement was defined as attending a minimum of two (50%) visits.
- Alcohol‐related admissions and all‐cause mortality post‐randomization, per arm, as reported in electronic health records.
- Self‐reported alcohol intake, obtained through timeline followback (Sobell & Sobell, 1996), is measured in units (1 unit = 8 g of ethanol).
- Alcohol Problems Questionnaire (APQ; Drummond, 1990).
- Severity of Alcohol Dependence Questionnaire (SADQ; Stockwell et al., 1979).
- Liver function (ascites, hepatic encephalopathy, jaundice, portal hypertension, upper gastrointestinal bleeding, variceal hemorrhage) and biomarkers (albumin; aspartate aminotransferase, AST; bilirubin; creatinine; gamma‐glutamyl transferase, GGT; international normalized ratio, INR; sodium).
- Model for End‐Stage Liver Disease 3.0 score (MELD 3.0 score; Kim et al., 2021).
- Child‐Pugh score, a validated model to assess prognosis in patients with cirrhosis (CPG; Pugh et al., 1973).

### Statistical analysis

SPSS Statistics version 29.0 was used to perform statistical analyses (IBM Corp, 2023).

Baseline sample characteristics were analyzed through descriptive statistics, and group differences were calculated (independent samples *t*‐test for continuous outcomes, Chi‐Square test for categorical outcomes).

Primary outcome data on feasibility (recruitment, randomization, study retention, completeness of the data, protocol fidelity) were reported through summary statistics, and additional analyses by arm were conducted where appropriate.

Given the nature of this pilot trial, hypothesis testing was not performed; per‐protocol analyses of secondary outcome data were carried out for exploratory purposes. Secondary outcome data (engagement, alcohol intake and liver outcomes) were reported through summary statistics, and additional analyses by arm were conducted where appropriate.

## RESULTS

### Baseline participant characteristics

The demographic profile, clinical history, and health services utilization of the sample are presented in Table 3. The mean age of the cohort was 48.47 (SD 10.08); 30% (*n* = 9) self‐identified as female and 70% (*n* = 29) as male. Regarding ethnicity, 13.30% (*n* = 4) identified as Asian, Asian British, or Asian Welsh, 6.70% (*n* = 2) as Black, Black British, Black Welsh, Caribbean, or African, and 80% (*n* = 24) as White, White British, or White Welsh. The demographic profile of participants enrolled in the pilot trial generally matches that of previous similar research, although the prevalence of participants of Asian, Asian British, or Asian Welsh ethnicity was higher (Mehta et al., 2024; Mellinger et al., 2023).

**TABLE 3:** Demographic profile, clinical history and health services utilization of the recruited sample.

Baseline characteristic	Overall sample (*n* = 30)	ILC only (*n* = 15)	ILC + CM (*n* = 15)	Group differences
Age (years), mean (SD, range); median	48.47 (10.08, 28–73); 46	50.27 (9.82, 36–73); 46	46.67 (10.34, 28–66); 46	*p* = 0.33
Sex assigned at birth, % (*n*)				
Female	30 (9)	33.30 (5)	26.70 (4)	*χ* ^2^(1) = 0.15, *p* = 0.69
Male	70 (21)	66.70 (10)	73.30 (11)	
Gender identity, % (*n*)				
Female	30 (9)	33.30 (5)	26.70 (4)	*χ* ^2^(1) = 0.15, *p* = 0.69
Male	70 (21)	66.70 (10)	73.30 (11)	
Ethnicity, % (*n*)				
Asian, Asian British or Asian Welsh	13.30 (4)	6.70 (1)	20 (3)	*χ* ^2^(2) = 1.16, *p* = 0.55
Black, Black British, Black Welsh, Caribbean or African	6.70 (2)	6.70 (1)	6.70 (1)	
White, White British or White Welsh	80 (24)	86.70 (12)	73.30 (12)	
Borough of residence, % (*n*)^a^				
Bexley	3.30 (1)	6.70 (1)	0 (0)	*χ* ^2^(5) = 3.60, *p* = 0.60
Croydon	3.30 (1)	0 (0)	6.70 (1)	
Lambeth	50 (15)	40 (6)	60 (9)	
Lewisham	6.70 (2)	6.70 (1)	6.70 (1)	
Southwark	30 (9)	40 (6)	20 (3)	
Wandsworth	6.70 (2)	6.70 (1)	6.70 (1)	
Marital status, % (*n*)				
Single	46.70 (14)	60 (9)	33.30 (5)	*χ* ^2^(4) = 4.34, *p* = 0.36
With partner	13.30 (4)	13.30 (2)	13.30 (2)	
Married	13.30 (4)	13.30 (2)	13.30 (2)	
Divorced	16.70 (5)	13.30 (2)	16.70 (5)	
Widowed	10 (3)	0 (0)	20 (3)	
Employment status, % (*n*)				
Employed	13.30 (4)	6.70 (1)	20 (3)	*χ* ^2^(3) = 2.38, *p* = 0.49
Self‐employed	10 (3)	6.70 (2)	13.30 (2)	
Not employed	63.30 (19)	66.70 (10)	60 (9)	
Retired	13.30 (4)	20.00 (3)	6.70 (1)	
Pre‐admission TLFB weekly intake (units), mean (SD, range); median	179.20 (70.22, 42–350); 180	174.40 (74.99, 42–350); 210	184.0 (67.45, 90–350); 140	*p* = 0.71
Pre‐admission TLFB units per drinking day, mean (SD, range); median	26.58 (10.14, 6–50); 30	25.98 (11.13, 6–50); 30	27.16 (9.41, 12.85–50); 30	*p* = 0.75
Pre‐admission percentage days abstinent, % mean (SD, range); median	1.42 (4.33, 0–14.20); 0	1.89 (4.99, 0–14.20); 0	0.94 (3.66, 0–14.20); 0	*p* = 0.55
APQ score, mean (SD, range); median^a^	16.16 (7.73, 3–30); 15.50	15.27 (7.39, 3–27); 16	16.92 (8.23, 6–30); 15	*p* = 0.06
SADQ score, mean (SD, range); median^a^	32.58 (16.65,8–60); 31.50	30.90 (18.35, 8–60); 29	34 (15.69, 8–58); 35	*p* = 0.66
ARLD diagnosis, % (*n*)^b^				
Non‐severe alcohol related hepatitis	6.70 (2)	6.70 (1)	6.70 (1)	*χ* ^2^(2) = 0.00, *p* = 1
Alcohol‐related cirrhosis	40 (12)	40 (6)	40 (6)	
Decompensated liver disease	53.30 (16)	53.30 (8)	53.30 (8)	
ARLD symptoms upon admission, % (*n*)^b^				
Ascites	33.30 (10)	26.70 (4)	40 (6)	*χ* ^2^(1) = 0.60, *p* = 0.43
Hepatic encephalopathy	16.70 (5)	13.30 (2)	20 (3)	*χ* ^2^(1) = 0.24, *p* = 0.62
Jaundice	16.70 (5)	20 (3)	13.30 (2)	*χ* ^2^(1) = 0.24, *p* = 0.62
Portal hypertension	23.30 (7)	26.70 (4)	20 (3)	*χ* ^2^(1) = 0.18, *p* = 0.66
Upper gastrointestinal bleeding	13.30 (4)	0 (0)	26.70 (4)	*χ* ^2^(1) = 4.61, *p* = 0.03
Variceal hemorrhage	30 (9)	20 (3)	40 (6)	*χ* ^2^(1) = 1.42, *p* = 0.23
Albumin (g/L), mean (SD, range); median	33.63 (7.91, 21–51); 33	33.13 (8.33, 23–47); 31	34.13 (7.73, 21–51); 34	*p* = 0.73
AST (U/L), mean (SD, range); median	106.53 (75.12,19–339); 79.50	90.33 (45.75, 41–201); 78	122.73 (95.04,19–339); 81	*p* = 0.24
Bilirubin (*μ*mol/L), mean (SD, range); median	94.23 (121.63, 6–476); 42.50	84.86 (118.5, 6–471); 42	103.60 (128.11, 6–476); 43	*p* = 0.68
Creatinine, (*μ*mol/L), mean (SD, range); median	61.16 (24.54, 31–128); 53	59.26 (18.21, 31–99); 457	63.06 (30.14, 32–128); 50	*p* = 0.68
GGT (U/L), mean (SD, range); median	616.27 (522.45, 83–2085); 448.50	623.73 (564.91, 83–2085); 1.10	608.80 (496.14, 135–1811); 423	*p* = 0.93
INR, mean (SD, range); median	1.22 (0.25, 0.90–2.11); 1.20	1.21 (0.29,0.90–2.11); 1.10	1.23 (0.22, 0.90–1.90); 1.20	*p* = 0.83
Sodium, (mmol/L), mean (SD, range); median	136.30 (5.01,122–142); 138	137.60 (4.42, 126–142); 139	135 (5.37, 122–140); 136	*p* = 0.15
MELD 3.0 score, mean (SD, range); median^c^	15.39 (6.50, 6–30); 14.50	14.42 (6.03, 7–26); 12.50	16.35 (7.03, 6–30); 16.50	*p* = 0.44
CPG score, mean (SD, range); median^c^	8.14 (1.89, 5–11); 8	8.64 (2.09, 5–11); 8.50	7.64 (1.59, 6–10); 7	*p* = 0.16
Mental health diagnoses, % (*n*)^b^				
Bipolar disorder	3.30 (1)	0 (0)	6.70 (1)	*χ* ^2^(1) = 1.03, *p* = 0.30
Generalized anxiety disorder	20 (6)	26.70 (4)	13.30 (2)	*χ* ^2^(1) = 0.08, *p* = 0.36
Major depressive disorder	56.70 (17)	73.30 (11)	40 (6)	*χ* ^2^(1) = 3.39, *p* = 0.06
Post‐traumatic stress disorder	10 (3)	6.70 (1)	13.30 (2)	*χ* ^2^(1) = 0.37, *p* = 0.54
History of extrahepatic comorbidity, % (*n*)^b^				
Asthma	10 (3)	6.70 (1)	13.30 (2)	*χ* ^2^(1) = 0.37, *p* = 0.54
Cancer	3.3 (1)	6.70 (1)	0 (0)	*χ* ^2^(1) = 1.03, *p* = 0.30
Chronic obstructive pulmonary disease	6.70 (2)	6.70 (1)	6.70 (1)	*χ* ^2^(1) = 0.00, *p* = 1
Diabetes	13.30 (4)	6.70 (1)	20 (3)	*χ* ^2^(1) = 1.15, *p* = 0.28
Hypertension	20 (6)	13.30 (2)	26.70 (4)	*χ* ^2^(1) = 0.83, *p* = 0.36
Multifactorial liver disease	3.30 (1)	0 (0)	6.70 (1)	*χ* ^2^(1) = 1.03, *p* = 0.30
Obesity	3.30 (1)	0 (0)	6.70 (1)	*χ* ^2^(1) = 1.03, *p* = 0.30
Organ failure	6.70 (2)	6.70 (1)	6.70 (1)	*χ* ^2^(1) = 0.00, *p* = 1
Acute kidney injury	6.70 (2)	0 (0)	13.30 (2)	*χ* ^2^(1) = 2.14, *p* = 0.14
Hepatitis C	16.70 (5)	20 (3)	13.30 (2)	*χ* ^2^(1) = 0.24, *p* = 0.62
Alcohol‐related admission, % (*n*)^b^	76.70 (23)	80 (12)	73.30 (11)	*χ* ^2^(1) = 0.18, *p* = 0.66
Length of admission, days (SD, range); median	14.47 (24.84, 2–127); 7	15.80 (31.02, 3–127); 7	13.13 (17.66, 4–74); 8	*p* = 0.77
Lifetime admissions, mean (SD, range); median	3.17 (3.69, 9–16); 2	2.53 (4.10, 0–16); 1	3.80 (3.25, 0–11); 4	*p* = 0.35
Contact with alcohol treatment and support pre‐admission, % (*n*)^b^				
Alcohol Assertive Outreach Team	26.70 (8)	26.70 (4)	26.70 (4)	*χ* ^2^(1) = 0.00, *p* = 1
CDAT	36.70 (11)	33.30 (5)	40 (6)	*χ* ^2^(1) = 1.44, *p* = 0.70
Peer support initiatives	23.30 (7)	26.70 (4)	20 (3)	*χ* ^2^(1) = 0.18, *p* = 0.66
Contact with ACT pre‐admission, % (*n*)^b^	80 (24)	73.30 (1)	86.70 (12)	*χ* ^2^(1) = 0.83, *p* = 0.36

*Note*: 1 unit = 8 g of ethanol. Independent samples *t*‐test calculated for continuous outcomes and Chi‐Square tests for categorical outcomes. Significance level *p* < 0.05.

Abbreviations: ACT, Alcohol Care Team; APQ, Alcohol Problems Questionnaire; ARLD, alcohol‐related liver disease; AST, aspartate aminotransferase; CDAT, community drug and alcohol treatment; CM, contingency management; CPG score, Child‐Pugh score; GGT, gamma‐glutamyl transferase; INR, international normalized ratio; KCH, King's College Hospital; MELD 3.0 score, Model for End‐Stage Liver Disease 3.0 score; *n*, number; SADQ, Severity of Alcohol Dependence Questionnaire; SD, standard deviation; TLFB, timeline followback.

^a^ Missing self‐reported data for *n* = 6 (ILC *n* = 4, ILC + CM *n* = 2).

^b^ Data obtained from electronic health records.

^c^ Calculated for participants presenting cirrhosis and decompensated liver disease.

Most participants (80%, *n* = 24) were known to the ACT from previous admissions (an average of 3 admissions pre‐randomization), but only a small proportion had engaged with community treatment services (36.70%, *n* = 11), peer support initiatives (23.30%, *n* = 7) or were supported by an assertive outreach (comprehensive, intensive delivery of community mental health care for service users who have not engaged effectively with treatment in the past) team (26.70%, *n* = 8). The median length of inpatient stay was 7 days (range 2–127).

In the month preceding admission, participants had an alcohol intake of 179.20 units per week (SD 70.22) and the mean percentage of days abstinent was 1.42% (SD 4.33). The mean APQ score was 16.16 (SD 7.52) and SADQ was 32.58 (SD 16.65), suggestive of severe alcohol dependence.

Over half of the participants (53.30%, *n* = 16) presented to the hospital with decompensated liver disease, 40% (*n* = 12) with cirrhosis, and 6.70% (*n* = 2) with non‐severe alcohol‐related hepatitis. Upon admission, 33.30% (*n* = 10) presented with ascites and 16.70% (*n* = 5) with jaundice. Hepatic encephalopathy was present in 16.70% (*n* = 5), presented with upper gastrointestinal bleeding in 13.30% (*n* = 4), and 30% (*n* = 9) with variceal hemorrhage, which are symptoms suggestive of portal hypertension. No participants had an active infection of hepatitis C at the time of recruitment. At baseline, MELD 3.0 and CPG scores were calculated for participants with cirrhosis and decompensated liver disease (*n* = 28) and had a median of 14.50 (range 6–30) and 8 (range 5–11) respectively. Additional data on liver biomarkers (albumin, AST, bilirubin, creatinine, GGT, INR, sodium) can be found in Table 3.

### Feasibility outcomes

The overall feasibility of the trial was evaluated as amber (when there are remediable aspects that need to be addressed before proceeding) in line with Avery's traffic light system.

#### Recruitment and study‐retention post‐randomization

**FIGURE 2:**
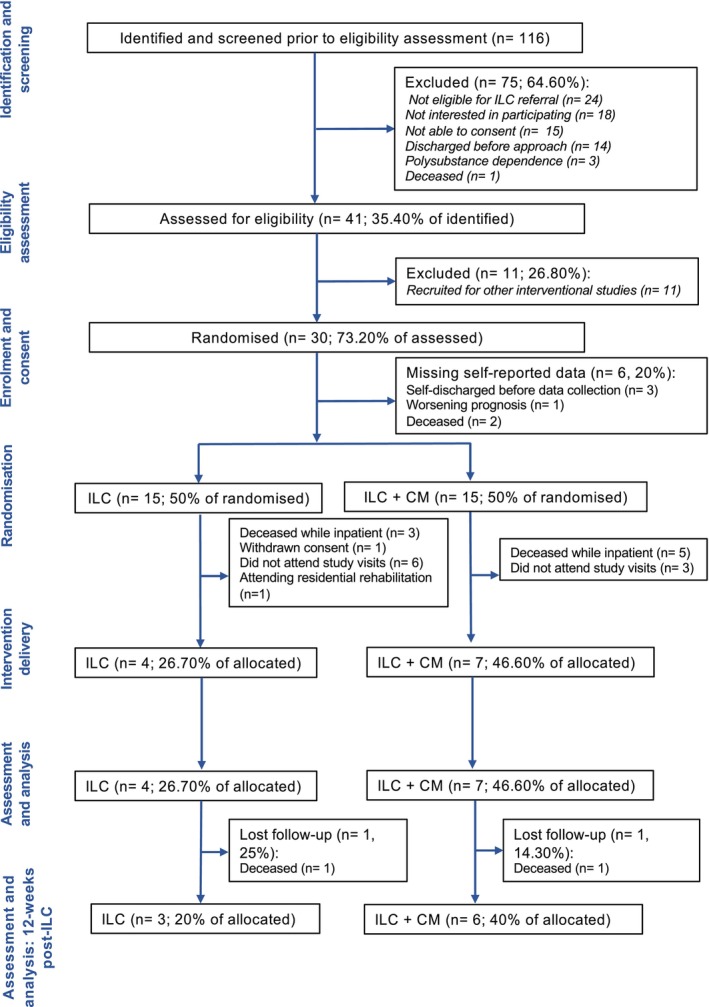
CONSORT flowchart.

Between January and December 2023, 116 patients (Figure 2) were identified for the pilot trial, and 41 were assessed for eligibility (eligibility rate 35.40%). Thirty patients provided informed consent (recruitment rate 73.20%); 15 were randomized to ILC only and 15 to ILC + CM. Following randomization, eight participants died in hospital before discharge (ILC *n* = 3, 20%; ILC + CM *n* = 5, 33.30%), highlighting the severity of this patient population; a further 11 did not engage. Overall, this accounted for a total loss of 19 participants (ILC *n* = 11, 73.30%; ILC + CM *n* = 8, 53.30%). One participant (*n* = 1, 6.60%) allocated to the control group withdrew from the study before receiving ILC. A total of 11 participants (*n* = 11; retention rate 36.70%) engaged with the trial. Of these, four (26.70%) received ILC only and seven (46.60%) received ILC + CM. Despite the small sample size, no differences in retention were observed between groups (*χ* ^2^(1) = 1.29, *p* = 0.25), although these values should be interpreted with caution. No severe adverse events related to the trial protocol occurred. In line with Avery's traffic light system, recruitment and study retention were considered amber (when there are remediable aspects that need to be addressed before proceeding). Reasons for suboptimal engagement with the trial and treatment have been reported in two separate qualitative investigations (Hemrage et al., 2024a, 2024b). In these investigations, a combination of interplay between individual, organizational, and structural factors, exacerbated by the COVID‐19 pandemic and socioeconomic landscape, was found to condition access to clinical research (Hemrage et al., 2024a). Additionally, the authors also found gaps in the care continuum, complex health needs, accessibility, and stigma to hinder treatment engagement among this clinical population (Hemrage et al., 2024b). For patients with comorbid AUD and ARLD, these barriers further contribute to a gap in effective evidence‐based treatment, exacerbating the health inequalities experienced by this clinical population.

#### Completeness of the data

The completeness of the data collected as part of the trial was satisfactory overall (green—no concerning aspects that need to be addressed before proceeding), and per protocol, the percentage of completed assessments being ≥80%. Differences in the completeness of data were observed between self‐reported outcomes and data extracted from electronic health records. At baseline, demographic and laboratory data extracted from electronic health records were available for all 30 participants (100% of completed planned assessments). Self‐reported data was available for 24 participants (80% of completed planned assessments). Reasons for missing data include self‐discharge after randomization (*n* = 3), worsening prognosis (*n* = 2) and death (*n* = 1). For participants self‐discharging, three contact attempts were made before being classified as lost to follow‐up. Alcohol intake, measured through TLFB, was available for all participants. TLFB was a self‐reported measure, but for participants with missing self‐reported data, alcohol intake was extracted from electronic health records.

For participants completing the trial (*n* = 11), the percentage of completed planned assessments at visit 4 was 100%. The baseline demographic characteristics, clinical history, and health services utilization for this subgroup are provided in Appendix S2. At the longest follow‐up (12‐weeks post‐ILC), two participants were deceased (ILC *n* = 1, ILC + CM *n* = 1). Therefore, self‐reported data were collected for nine participants. 88.80% (8/9) of planned laboratory assessments were conducted at the longest follow‐up; data for one participant was missing due to an IT incident with the laboratory provider, which prevented the timely processing of additional blood samples, but this did not pose any risks to patient data confidentiality.

#### Protocol fidelity

This criterion was considered amber (when there are remediable aspects that need to be addressed before proceeding). From the 11 participants (*n* = 11) completing the study, the mean number of days between discharge and the first visit was 50.90 (SD 38.86) and the median was 37. Six participants (*n* = 6) attended their first appointment within the planned period (14 ± 7). One participant (*n* = 1) was re‐admitted twice before receiving ILC; this accounted for an 88‐day deviation from the protocol. Another participant (*n* = 1) attended a residential rehabilitation program following discharge and was therefore not able to receive the outpatient intervention offered as part of the trial. This resulted in a 103‐day deviation. The observed duration of intervention delivery had a mean of 123.54 (SD 63.09) and a median of 98 days.

Protocol fidelity was susceptible to challenges inherent to implementing an experimental protocol within a routine clinical setting. Factors such as slot and clinic capacity, clinic cancellation due to industrial action, and the trust‐wide implementation of a new electronic system resulted in delays in offering ILC appointments. Notwithstanding these challenges, all participants in the CM group received CM upon attending the ILC clinic. CM provision, managed by the research team, was nonburdensome and effective. No acceptability or practical concerns were raised by the ILC consultants or participants regarding voucher‐based CM.

### Exploratory outcomes

#### Engagement, alcohol‐related admissions and mortality

A trending higher percentage of participants in the CM group engaged with ILC (ILC 26.70%, ILC + CM 46.70%). Two participants in the CM group returned to drinking following discharge. Higher admissions were observed in the CM group (ILC + CM = 2.14, SD 2.73, range 0–6; ILC = 1, SD 2, range 0–6). Two participants were deceased while in the trial, corresponding to 25% (*n* = 1) in the control group and 14.30% (*n* = 1) in the CM group.

#### Alcohol use

**FIGURE 3:**
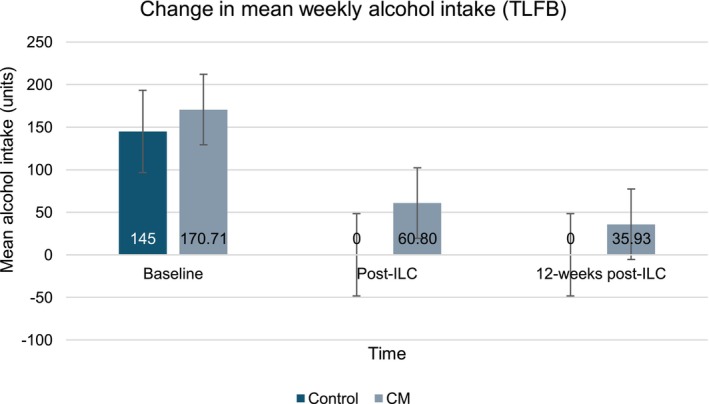
Change in mean weekly alcohol intake (measured TLFB). CM, contingency management; ILC, integrated liver care.

Across both groups, the overall weekly alcohol intake had a 76% reduction trend to 38.69 units (SD 105.71, range 0–350) post‐ILC. Specifically, participants in the control group were abstinent, and participants in the CM group reduced their intake by 64.30%, with an intake of 60.80 units (SD 130.60). Figure 3 provides a graphical representation of the change in weekly alcohol intake.

At the longest follow‐up, an 85.90% (23.95 units, SD 50.18) reduction trend on the weekly was observed compared to baseline. Participants in the control group sustained abstinence, and those receiving CM showed a 78.80% decreasing trend compared to baseline (35.93 units, SD 59.27). A corresponding trending increase in the percentage of days abstinent was observed for both groups, which was higher in the control group (100%) compared to CM (75.48% post‐ILC, 71.40% 12‐weeks post‐ILC).

Across both groups, trending reductions in APQ and SADQ scores were observed post‐ILC and at the longest follow‐up. Within the APQ, improvements were mostly noted in the social, physical, and psychological domains, suggesting that engagement with ILC may support the management of AUD and ARLD. At the longest follow‐up, a trend for improved SADQ scores was noted across both groups (ILC = 0.33, SD 0.57; ILC + CM = 11, SD 10.78). Given the increasing trend for abstinence observed in the control arm, the reduction in SADQ scores was less visible in the CM arm. Summary measures of alcohol outcomes are presented in Table 4.

**TABLE 4:** Alcohol use outcomes for participants completing the pilot trial (*n* = 11).

Alcohol use outcomes	Baseline (*n* = 11)				Post‐ILC (*n* = 11)				12 Weeks post‐ILC (*n* = 9)			
	Overall (*n* = 11)	Control (*n* = 4)	CM (*n* = 7)	Group difference	Overall (*n* = 11)	Control (*n* = 4)	CM (*n* = 7)	Group difference	Overall (*n* = 9)	Control (*n* = 3)	CM (*n* = 6)	Group difference
TLFB weekly units, mean (SD, range); median	161.36 (74.36, 80–350); 140	145 (53.22, 80–210); 145	170.71 (86.71, 90–350); 140	*p* = 0.55	38.69 (105.71, 0–350); 0	0 (0); 0	60.80 (130.60, 0–350); 0	*p* = 0.38	23.95 (50.18,0–75.60); 0	0 (0); 0	35.93 (59.2, 0–75.60); 0	*p* = 0.34
TLFB units per drinking day, mean (SD, range); median	25.72 (11.38, 11.40–50); 20	25.98 (11.13, 6–50); 25	27.17 (9.41, 12.85–50); 30	*p* = 0.83	5.52 (15.10, 0–50); 0	0 (0); 0	8.68 (18.65, 0–50); 0	*p* = 0.26	3.42 (7.16, 0–20)	0 (0); 0	5.13 (8.46, 0–20)	*p* = 0.19
Percentage days abstinent, % mean (SD, range); median	2.58 (5.74, 0–14.20); 0	1.89 (4.99, 0–14.20); 0	0.94 (3.66, 0–14.20); 0	*p* = 0.72	84.40 (35.28, 0–100); 100	100 (0, 100); 100	75.48 (42.66, 0–100); 0	*p* = 0.17	80.93 (38.49, 0–100); 100	100 (0, 100); 100	71.40 (45.20, 0–100); 100	*p* = 0.18
Return to any drinking, % (*n*)	—	—	—	—	18.20 (2)	0 (0)	28.60 (2)	*χ* ^2^(1) = 1.397, *p* = 0.23	22.20 (2)	0 (0)	33.30 (2)	*χ* ^2^(1) = 1.286, *p* = 0.25
Abstinence, % (*n*)	—	—	—	—	81.80 (9)	100 (4)	71.40 (5)	*χ* ^2^(1) = 1.397, *p* = 0.23	77.80 (7)	100 (0)	66.70 (4)	*χ* ^2^(1) = 1.286, *p* = 0.25
APQ score, mean (SD, range); median	18.81 (8.54,4–30); 18	13 (7.39, 4–22); 13	22.14 (7.66, 10–30); 24	*p* = 0.08	5.81 (6.16,0–21); 4	4.25 (1.89,3–7); 3.50	6.71 (7.67, 0–21); 5	*p* = 0.55	4.55 (5.83, 0–18); 1	3.66 (4.61, 1–9); 1	5 (6.72, 0–18); 3	*p* = 0.77
SADQ score, mean (SD, range); median	19.72 (14.73, 0–50); 18	10 (9.55,0–21); 8.50	25.28 (14.73,8–50); 19	*p* = 0.09	9.09 (12.07,0–39); 6	5.25 (5.12,0–12); 4.50	11.28 (14.64,0–39); 6	*p* = 0.45	7.44 (10.06,0–26); 1	0.33 (0.57, 0–1); 0	11 (10.78,0–26); 10	*p* = 0.14

Abbreviations: APQ, Alcohol Problems Questionnaire; CM, contingency management; ILC, integrated liver care; *n*, number; SADQ, Severity of Alcohol Dependence Questionnaire; SD, standard deviation; TLFB, timeline followback.

#### Liver function and biomarkers

Compared to baseline, the proportion of participants presenting with signs of ascites improved over time, observed in three post‐ILC (ILC *n* = 1, ILC + CM *n* = 2) and in two participants (ILC *n* = 1, ILC + CM *n* = 1) at 12 weeks post‐ILC. No participants presented with hepatic encephalopathy following discharge.

**FIGURE 4:**
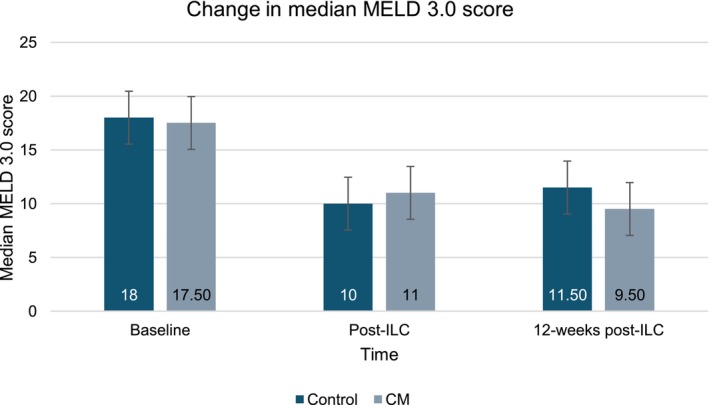
Change in median MELD 3.0 score.

**FIGURE 5:**
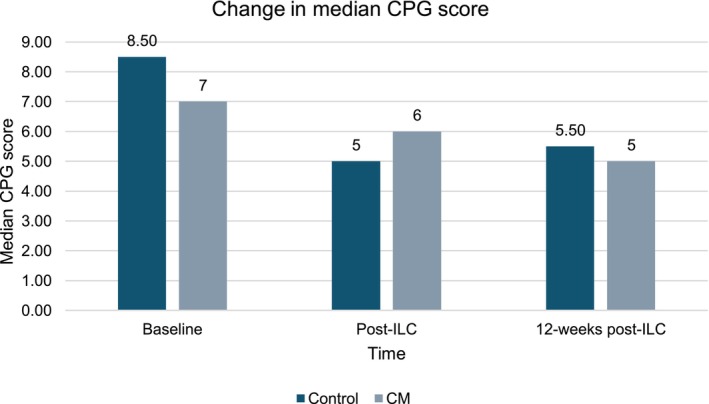
Change in median CPG score.

Among those completing the trial (*n* = 11), a trend for improvements on the MELD 3.0 and CPG scores was observed over time (Figures 4 and 5). The median MELD 3.0 decreased from 18 (range 3–6) to 9.50 (range 7–14) at the longest follow‐up. The median CPG score improved from 7 (range 5–11) to 5 (range 5–7), representing a shift from Class B to Class A.

Summary measures of additional data on liver biomarkers can be found in Table 5.

**TABLE 5:** Liver health outcomes for participants completing the pilot trial (*n* = 11).

Liver function and biomarkers	Baseline (*n* = 11)				Post‐ILC (*n* = 11)				12 Weeks post‐ILC (*n* = 9)			
	Overall (*n* = 11)	Control (*n* = 4)	CM (*n* = 7)	Group difference	Overall (*n* = 11)	Control (*n* = 4)	CM (*n* = 7)	Group difference	Overall (*n* = 9)	Control (*n* = 3)^a^	CM (*n* = 6)	Group difference
Albumin (g/L), mean (SD, range); median	35.63 (8.02, 27–51); 34	37 (9.52, 27–47); 37	34.85 (7.75, 27–47); 34	*p* = 0.69	40.54 (7.52, 26–53); 41	41 (2.16, 38–43); 41.5	40.28 (9.58, 26–53); 40	*p* = 0.85	45.62 (5.06, 37–53); 45	47 (5.65, 43–51); 47	45.16 (5.34, 37–53); 45	*p* = 0.69
AST (U/L), mean (SD, range); median	114.72 (99.06, 4–339); 81	73.5 (52.44, 4–128); 81	138.28 (114.89, 19–339); 81	*p* = 0.32	57.27 (44.52, 22–158); 38	36.25 (11.84, 22–46); 38.50	69.28 (52.64, 35–158); 38	*p* = 0.25	92.87 (74.37, 34–211); 59	53 (2.82, 51–55); 53	106.16 (83.02, 34–211); 72	*p* = 0.42
Bilirubin (*μ*mol/L), mean (SD, range); median	114.36 (106.717,6–314); 64	129 (134.95, 6–314); 98	106 (98.23, 6–282); 64	*p* = 0.75	47.81 (78.55, 5–276); 64	24.75 (13.57, 5–35); 29.50	61 (98.16, 8–276); 20	*p* = 0.49	16.87 (11.11, 6–34); 26	14.50 (12.02, 6–23); 14.50	17.66 (11.877, 6–34); 12.50	*p* = 0.75
Creatinine, (*μ*mol/L), mean (SD, range); median	58.81 (15.35, 38–91); 53	63.25 (12.60, 45–73); 67.50	56.28 (17.11, 38–91); 50	*p* = 0.49	66.54 (22.25, 36–106); 67	76 (20.21, 62–106); 68	61.14 (22.95, 35–98); 56	*p* = 0.31	79.25 (21.15, 52–103); 78	103 (0); 103	71.33 (18.05, 52–95); 63	*p* = 0.05
GGT (U/L), mean (SD, range); median	872.45 (546.29, 114–1804); 536	519.25 (426, 114–1108); 553	1074.28 (523.32, 381–1804); 436	*p* = 0.10	314 (219.30, 56–791); 272	193.50 (162.87, 56–418); 150	382.85 (227.48, 152–791); 277	*p* = 0.18	491.62 (241.418, 172–969); 416	502.50 (232.63, 338–667); 502.50	488 (265.91, 72–969); 416	*p* = 0.94
INR, mean (SD, range); median	1.22 (0.14, 1–1.4); 1.20	1.25 (0.17, 1.1–1.4); 1.25	1.21 (0.14, 1–1.40); 1.20	*p* = 0.74	1.16 (0.17, 1–1.5); 1.10	1.12 (0.09, 1–1.2); 1.15	1.03 (0.08, 1–1.50); 1.10	*p* = 0.60	1.05 (0.75, 1–1.20); 1	1.10 (0); 1.10	1.03 (0.08, 1–1.20); 1.10	*p* = 0.31
Sodium (mmol/L), mean (SD, range); median	135.81 (6.03, 123–142); 138	136.75 (7.27, 126–142); 139.50	135.28 (5.86, 123–140); 137	*p* = 0.36	137.81 (4.21, 130–145); 139	136.50 (4.50, 130–140); 138	138.58 (4.19, 132–145); 139	*p* = 0.23	136.87 (5.40, 130–143); 137	135.50 (6.36, 131–140); 135.50	137.33 (5.64, 130–143); 138	*p* = 0.71
Ascites, % (*n*)	63.60 (7)	75 (3)	57.10 (4)	*χ* ^2^(1) =0.35, *p* = 0.50	27.30 (3)	25 (1)	28.60 (2)	*χ* ^2^(1) = 0.01, *p* = 0.89	22.20 (2)	33 (1)	16.70 (1)	*χ* ^2^(1) =0.32, *p* = 0.57
Hepatic encephalopathy, % (*n*)	36.40 (4)	25 (1)	42.90 (3)	*χ* ^2^(1) = 0.35, *p* = 0.55	—	—	—	—	—	—	—	—
MELD 3.0 score, mean (SD, range); median^b^	17.72 (7.21, 3–6); 18	17.50 (7.5, 9–26); 18	17.85 (7.64, 6–30); 17.50	*p* = 0.94	11.54 (5.27, 6–24); 11	11 (3.55, 8–16); 10	11.85 (6.30, 6–24); 11	*p* = 0.81	10.12 (2.16, 7–14); 9.50	11.50 (3.52, 9–15); 11.50	9.66 (1.75, 7–12); 9.50	*p* = 0.33
CPG score, mean (SD, range); median^b^	8.27 (2.10, 5–11); 7	8.75 (2.62, 5–11); 8.50	8 (1.91, 6–10); 7	*p* = 0.59	6 (1.09, 5–8); 6	5.50 (1, 5–7); 5	6.28 (1.11, 5–8); 6	*p* = 0.27	5.37 (0.74, 5–7); 5	5.5 (0.70, 5–6); 5.50	5.33 (0.81, 5–7); 5	*p* = 0.80

*Note*: Independent samples *t*‐test calculated for continuous outcomes and Chi‐Square tests for categorical outcomes. Significance level *p* < 0.05.

Abbreviations: AST, aspartate aminotransferase; CM, contingency management; CPG score, Child‐Pugh score; GGT, gamma‐glutamyl transferase; INR, international normalized ratio; MELD score, Model for End‐Stage Liver Disease 3.0 score; *n*, number; SD, standard deviation.

^a^ Data missing for one participant (*n* = 1).

^b^ Calculated for participants presenting cirrhosis and decompensated liver disease.

## DISCUSSION

This pilot trial explored the feasibility of delivering voucher‐based CM to promote engagement with ILC. Through its adaptive design, this research allowed for identifying and addressing practicalities encountered while implementing the experimental protocol in a real‐world clinical setting. While challenges associated with trial performance were encountered, the role of CM to promote engagement and subsequently improve patient outcomes appears promising.

The primary outcomes of the trial included the evaluation of feasibility criteria (recruitment, retention, completeness of data and protocol fidelity). In line with the traffic light system proposed by Avery, three feasibility criteria (recruitment, study retention post‐randomization, protocol fidelity) were amber, and one (completeness of data) was green. The overall performance of the trial was evaluated as amber, suggesting that there are remediable aspects that need to be modified before proceeding to the next phases of research.

During the initial stages of study planning, the recruitment period was set to have an initial duration of 12 weeks (at a planned recruitment rate of 2–3 participants per week). However, the observed recruitment rates were lower than anticipated; this was also the case in a similar pilot trial, in which a lower recruitment rate was observed within an inpatient setting compared to an outpatient setting (Mellinger et al., 2023). An amendment was sought to extend the recruitment period and add two additional recruitment sites, but this had little effect on improving recruitment. The recruitment rate was 73.20%, and the retention rate was 36.70%. The low retention rate suggests this clinical population faces substantial barriers to accessing health‐enabling resources (Hemrage et al., 2024a, 2024b). Accordingly, attrition among clinical populations presenting AUD and associated comorbidity has been widely described across health research (Comerford et al., 2017; Weinrieb et al., 2001).

While the UK has a free, publicly funded healthcare system, participants declined participation due to the treatment schedule and research burden. Mainly, this was because the ILC visits took place on a working day, and participants were required to attend the clinic during working hours. Complementary routes to engagement, such as digital health technologies, could therefore mediate patient‐led, meaningful engagement in research and improve access to health‐enabling resources (Mehta et al., 2024; Mellinger et al., 2023). Adopting such approaches could improve recruitment, retention, and protocol fidelity, while reducing the overall burden of research and treatment both on patients and healthcare providers.

Enrolment into the study, baseline data collection, and randomization took place simultaneously. These procedures were conducted during inpatient admission, and eight participants (*n* = 8, 26.70%) were deceased before being discharged. This high mortality rate, observed in the inpatient setting, had implications for overall retention in the trial, as this group of patients was recruited but did not receive the intervention. Trial performance would therefore benefit from randomizing participants at the point of discharge or first outpatient appointment instead.

CM provision was considered effective and deliverable, and all participants in the CM group received the intervention as planned. However, there were challenges associated with delivering the ILC in real‐world clinical settings. Accommodating additional patients as part of the trial and providing sessions per the protocol schedule was resource‐intensive for the service given the impact of healthcare worker strikes, an IT incident, and the implementation of a new electronic healthcare record system. All participants completing the study received ILC, but its delivery took longer due to service capacity, clinical backlogs, and caseload prioritization. This consisted of a major source of protocol deviation. Consequently, addressing such barriers could enable prompt treatment following discharge. Notwithstanding the challenges listed above, engagement with ILC was shown to be a feasible, measurable target behavior for CM. Since study participation was measured through attendance at ILC, primary outcome data was easily obtained from electronic health records. Exploratory outcomes were also feasible to collect and analyze.

Sustained engagement with integrated treatment in ARLD has the potential to improve patient outcomes (DiMartini et al., 2022; Mellinger et al., 2023; Winder et al., 2022). Although the trial was not designed or powered to detect statistically significant changes, exploratory data among those with follow‐up data demonstrated a favorable trend in alcohol intake and liver function outcomes with ILC engagement. The findings suggest that sustained treatment engagement may improve patient outcomes in comorbid AUD and ARLD and that adjunctive CM can support sustained engagement. While ILC, through the delivery of additional psychosocial and pharmacological interventions, may have diluted the effect of CM, a trend for higher rates of engagement was observed in the CM group. On the other hand, in the control group, an improving trend in terms of the percentage of days abstinent compared to the CM group was observed. However, given the small sample, it is not possible to establish a correlation between this outcome and the intervention. Therefore, larger studies could further ascertain the clinical significance of providing CM to this clinical population.

### Strengths and limitations

Embedding the experimental protocol in a clinical setting enabled an organic understanding of real‐world implementation and delivery of voucher‐based CM, which is a strength of this research. Particularly, this allowed the identification of factors that shape research design and those inherent to care delivery in an under‐served clinical population and can inform future trials aimed at developing novel interventions for this patient group.

In this pilot trial, CM was delivered as an adjunctive to ILC. No previous research has attempted to deliver CM to promote engagement with multidisciplinary ARLD care (Hemrage et al., 2023; Khan et al., 2016). ILC has been increasingly recommended to bridge the treatment gap in comorbid AUD and ARLD (DiMartini et al., 2022; Haque & Leggio, 2024; Singal et al., 2024). The integration of hepatology and addiction psychiatry in study design has also been recommended to generate higher quality research in this area (Lee et al., 2024).

From a healthcare provider perspective, pragmatic barriers to the wider uptake and implementation of CM in clinical settings include ensuring the supply of incentives and adapting standard clinical workflow to deliver CM (Sinclair et al., 2011). The administrative aspects related to CM delivery were handled by the research team, which circumvented these barriers to implementation. Attendance‐based CM is also logistically simpler and less resource‐intensive compared to abstinence‐based CM. For the management of clinically heterogeneous populations, attendance‐based CM can serve as an adjunct to treatment matching and person‐centered care (Hell et al., 2021; Witkiewitz et al., 2010). This can enable a more tailored, individual approach to treatment compared to abstinence‐focused interventions.

This research also had some limitations. The first limitation relates to the demographic profile of the cohort and its representativeness. Most participants self‐identified as male and were of White, White British, or White Welsh ethnicity. This may impact the generalizability of the findings. Several lines of evidence have highlighted gender and ethnic differences in ARLD prevalence, severity, treatment access, and mortality (Levy et al., 2015; McElroy et al., 2020; Mellinger et al., 2019; Niu et al., 2023; Probst et al., 2015). Addressing these variations is key for the development of person‐centered approaches tailored to subgroups that are more susceptible to alcohol‐related harm. Additionally, data on socioeconomic status was not collected; this was aimed at minimizing the research burden upon participants. Socioeconomic status is a known modifier of alcohol consumption, harm, and ARLD incidence (Askgaard et al., 2024; Probst et al., 2020; Roblero et al., 2021). Therefore, it could be relevant to consider measurements of socioeconomic status to inform the development of approaches mitigating health inequalities across sociodemographic groups within the context of comorbid AUD and ARLD.

As a single‐centre study, the findings may not extend to all service users with comorbid AUD and ARLD across the UK or internationally. This is particularly relevant given the geographical variation between areas of high prevalence of ARLD compared to research hotspots in the UK (Tavabie et al., 2024). Participants were also recruited from an inpatient setting. This may prevent the understanding of treatment experience and health‐promoting behavior among service users who are not formally admitted to the hospital and those who do not voluntarily engage with services. In addition, a longer timeframe would allow for a better understanding of CM's long‐term impact on health‐promoting behavior. Many service users return to drinking within 6 months of admission, and multiple attempts at recovery might be needed before meaningful treatment engagement and improvements in alcohol intake and liver function outcomes are noticeable (Green et al., 2023). In addition, exploratory data is reflective only of those participants engaging with the trial, so the findings need to be interpreted with caution. As a result, multi‐centre studies with longer follow‐up times are needed to further ascertain the clinical significance of CM to improve patient outcomes in comorbid AUD and ARLD.

## CONCLUSION

The evidence generated in this pilot trial is of practical significance in advancing treatment in comorbid AUD and ARLD, and the scope of CM appeared promising in this clinical population. The pilot trial allowed the identification of study aspects that need to be addressed when planning for future research (recruitment, study retention post‐randomization, protocol fidelity). A trend for increased engagement was observed among participants receiving CM, suggesting its potential to enable health‐promoting behavior. The adopted procedure for CM delivery was feasible to implement. In turn, a trend for reduced alcohol intake and improved liver health outcomes was observed with participants engaging in ILC treatment engagement. Future research could explore the integration of digital health approaches to support the scalability of the research while fostering continuity of care for this clinical population.

## AUTHOR CONTRIBUTIONS

SH: conceptualization, methodology, formal analysis, data curation, investigation, writing – original writing, review, and editing; NK: investigation, writing – review and editing; NS: investigation, writing – review and editing; SP: conceptualization, supervision, writing – review and editing; PD: conceptualization, supervision, writing – review and editing; CD: conceptualization, supervision, writing – review and editing.

## FUNDING INFORMATION

This work was supported by the National Institute for Health Research Applied Research Collaboration South London (grant number NIHR200152). The views expressed are those of the authors and not necessarily those of the NIHR or the Department of Health and Social Care. CD, PD, and NK were supported by the NIHR Specialist Biomedical Research Centre for Mental Health at South London and Maudsley NHS Foundation Trust, King's College London. CD and PD were also supported by the NIHR Collaboration for Leadership in Applied Health Research and Care at King's College Hospital NHS Foundation Trust and the National Institute for Health Research (NIHR) Applied Research Collaboration South London (NIHR ARC South London) at King's College Hospital NHS Foundation Trust. CD was supported by an NIHR Senior Investigator Award.

## CONFLICT OF INTEREST STATEMENT

In the last four years, SP has been part‐funded by income from research grants obtained from MundiPharma Research Ltd. and Camurus AB. The remaining authors declare that they have no competing interests.

## ETHICS STATEMENT

The pilot study has been reviewed by King's College London and King's College Hospital according to the research ethical standards in place. A favorable ethical opinion was granted by Camden and Kings Cross NHS Research Ethics Committee (reference 22/LO/0744). Written informed consent was obtained from all participants.

## Supporting information


Appendix S1

Appendix S2

## Data Availability

The data that support the findings of this study are available on request from the corresponding author. The data are not publicly available due to privacy or ethical restrictions.
