# Supplementary material for: Contingency management to promote treatment engagement in comorbid alcohol use disorder and alcohol‐related liver disease: Findings from a pilot randomized controlled trial
Source: Alcohol Clin Exp Res (Hoboken). 2025 Mar 10;49(4):893–910. doi: 10.1111/acer.70018 (PMC12012876; doi:10.1111/acer.70018)
Supplement: Supplementary file 2 — Appendix S2 [file ACER-49-893-s001.docx]

Appendix S2: Demographic profile, clinical history and health services utilisation for participants completing the pilot trial.

| **Baseline characteristic** | **Completed (n=11)** | **Control group (n=4)** | **CM group (n=7)** | **Group differences** |
| --- | --- | --- | --- | --- |
| Age (years), mean (SD, range); median | 43.64 (9.15, 28-60); 46 | 44.75 (6.29, 36-51); 46 | 43 (10.89, 28-60); 41 | p=0.77 |
| Sex assigned at birth, % *(n)* |  |  |  |  |
| Female | 9.10 (1) | - | 14.30 (1) | χ^2^(1)=0.62, p=0.42 |
| Male | 90.90 (10) | 100 (4) | 85.70 (6) |  |
| Gender identity, % *(n)* |  |  |  |  |
| Female | 9.10 (1) | - | 14.30 (1) | χ^2^(1)=0.62, p=0.42 |
| Male | 90.90 (10) | 100 (4) | 85.70 (6) |  |
| Ethnicity, % *(n)* |  |  |  |  |
| Asian, Asian British or Asian Welsh | 18.20 (2) | 25 (1) | 14.30 (1) | χ^2^ (1)=0.19, p=0.65 |
| White, White British or White Welsh | 81.80 (9) | 75 (3) | 85.70 (6) |  |
| Borough of residence, % *(n)*^a^ |  |  |  |  |
| Croydon | 9.10 (1) | - | 6.70 (1) | χ^2^ (3)=2.57, p=0.46 |
| Lambeth | 36.40 (4) | 25 (1) | 60 (9) |  |
| Lewisham | 9.10 (1) | - | 6.70 (1) |  |
| Southwark | 45.50 (5) | 75 (3) | 20 (3) |  |
| Marital status, % *(n)* |  |  |  |  |
| Single | 36.40 (4) | 50 (2) | 28.60 (2) | χ^2^ (3)=2.35, p=0.50 |
| With partner | 18.20 (2) | 25 (1) | 14.30 (1) |  |
| Married | 18.20 (2) | 25 (1) | 14.30 (1) |  |
| Divorced | 27.30 (3) | - | 42.90 (3) |  |
| Employment status, % *(n)* |  |  |  |  |
| Employed | 18.20 (2) | - | 28.60 (2) | χ^2^ (3)=3.59, p=0.16 |
| Self-employed | 18.20 (2) | - | 28.60 (2) |  |
| Not employed | 63.60 (7) | 100 (4) | 42.90 (3) |  |
| Pre-admission TLFB weekly intake (units), mean (SD, range); median | 161.36 (74.36, 80-350); 140 | 145 (53.22, 80-210); 145 | 170.71 (86.71, 90-350); 140 | p=0.55 |
| TLFB units per drinking day, mean (SD, range); median | 25.72 (11.38, 11.40-50); 20 | 25.98 (11.13, 6-50); 25 | 27.17 (9.41, 12.85-50); 30 | p=0.83 |
| Percentage days abstinent, % mean (SD, range); median | 2.58 (5.74, 0-14.20); 0 | 1.89 (4.99, 0-14.20); 0 | 0.94 (3.66, 0-14.20); 0 | p=0.72 |
| APQ score, mean (SD, range); median | 18.81 (8.54,4-30); 18 | 13 (7.39, 4-22); 13 | 22.14 (7.66, 10-30); 24 | p=0.08 |
| SADQ score, mean (SD, range); median | 19.72 (14.73, 0-50); 18 | 10 (9.55,0-21); 8.50 | 25.28 (14.73, 8-50); 19 | p=0.09 |
| ARLD diagnosis, % *(n)*^a^ |  |  |  |  |
| Alcohol-related cirrhosis | 36.40 (4) | 25 (1) | 42.90 (3) | χ^2^(1) =0.35, p=0.55 |
| Decompensated liver disease | 63.60 (7) | 75 (3) | 57.10 (4) |  |
| ARLD symptoms upon admission, % *(n)*^a^ |  |  |  |  |
| Ascites | 63.60 (7) | 75 (3) | 57.10 (4) | χ^2^(1) =0.35, p=0.55 |
| Hepatic encephalopathy | 36.40 (4) | 25 (1) | 43.90 (3) | χ^2^(1) =0.35, p=0.55 |
| Jaundice | 27.30 (3) | 25 (1) | 28.60 (2) | χ^2^(1) =0.01, p=0.89 |
| Portal hypertension | 27.30 (3) | 50 (2) | 14.30 (1) | χ^2^ (1)=1.63, p=0.20 |
| Upper gastrointestinal bleeding | 18.20 (2) | - | 28.60 (2) | χ^2^ (1)=1.39, p=0.23 |
| Variceal haemorrhage | 27.30 (3) | - | 42.90 (3) | χ^2^ (1)=2.35 p=0.12 |
| Albumin (g/L), mean (SD, range); median | 35.63 (8.02, 27-51); 34 | 37 (9.52, 27-47); 37 | 34.85 (7.755, 27-47); 34 | p=0.69 |
| AST (U/L), mean (SD, range); median | 114.72 (99.06, 4-339); 81 | 73.50 (52.44, 4-128); 81 | 138.28 (114.89, 19-339); 81 | p=0.32 |
| Bilirubin (μmol/L), mean (SD, range); median | 114.36 (106.717, 6-314); 64 | 129 (134.95, 6-314); 98 | 106 (98.23, 6-282); 64 | p=0.75 |
| Creatinine, (μmol/L), mean (SD, range); median | 58.81 (15.35, 38-91); 53 | 63.25 (12.60, 45-73); 67.5 | 56.28 (17.11, 38-91); 50 | p=0.49 |
| GGT (U/L), mean (SD, range); median | 872.45 (546.29, 114-1804); 536 | 519.25 (426, 114-1108); 553 | 1074.28 (523.32, 381-1804); 436 | p=0.10 |
| INR, mean (SD, range); median | 1.22 (0.14, 1-1.4); 1.2 | 1.25 (0.17,1.1-1.4); 1.25 | 1.21 (0.14, 1-1.40); 1.2 | p=0.74 |
| Sodium, (mmol/L), mean (SD, range); median | 135.81 (6.03, 123-142); 138 | 136.75 (7.27, 126-142); 139.5 | 135.28 (5.86, 123-140); 137 | p=0.36 |
| MELD 3.0 score, mean (SD, range)^b^; median | 17.72 (7.21, 3-6); 18 | 17.50 (7.5, 9-26); 18 | 17.85 (7.64, 6-30); 17.5 | p=0.94 |
| CPG score, mean (SD, range)^b^; median | 8.27 (2.10, 5-11); 7 | 8.75 (2.62, 5-11); 8.5 | 8 (1.91, 6-10); 7 | p=0.59 |
| Mental health diagnoses, % *(n)*^a^ |  |  |  |  |
| Generalised anxiety disorder | 9.1 (1) | - | 14.30 (1) | χ^2^ (1)=0.62, p=0.42 |
| Major depressive disorder | 45.5 (5) | 50 (2) | 42.90 (3) | χ^2^ (1)=0.05, p=0.81 |
| Post-traumatic stress disorder | 18.2 (2) | 25 (1) | 14.30 (1) | χ^2^ (1)=0.19, p=0.65 |
| History of extrahepatic comorbidity, % *(n)*^a^ |  |  |  |  |
| Chronic obstructive pulmonary disease | 9.10 (1) | - | 14.30 (1) | χ^2^ (1)=0.62, p=0.42 |
| Diabetes | 9.10 (1) | - | 14.30 (1) | χ^2^ (1)=0.62, p=0.42 |
| Hypertension | 18.20 (2) | 25 (1) | 14.30 (1) | χ^2^ (1)=0.19, p=0.65 |
| Organ failure | 18.20 (2) | 25 (1) | 14.30 (1) | χ^2^ (1)=0.19, p=0.65 |
| Acute kidney injury | 18.20 (2) | - | 28.60 (2) | χ^2^ (1)=1.92, p=0.16 |
| Hepatitis C | 9.10 (1) | 25 (1) | - | χ^2^ (1)=0.24 p=0.62 |
| Alcohol-related admission, % *(n)*^a^ | 72.70 (8) | 50 (2) | 85.70 (6) | χ^2^ (1)=1.63, p=0.20 |
| Length of admission, days (SD, range); median | 8 (5.29, 4-19); 5 | 15.80 (31.02, 3-127); 5.50 | 13.13 (17.66, 4-74); 4 | p=0.77 |
| Lifetime admissions, mean (SD, range) | 1.64 (2.37, 0-7); 1 | 2.53 (4.10, 0-16); 0 | 3.80 (3.25, 0-11); 1 | p=0.35 |
| Contact with alcohol treatment and support pre-admission, % *(n)*^b^ |  |  |  |  |
| Alcohol Assertive Outreach Team | 18.20 (2) | - | 28.60 (2) | x^2^(1)=1.39, p=0.23 |
| CDAT | 45.50 (5) | 50 (2) | 42.90 (3) | x^2^(1)=0.05, p=0.81 |
| Peer support initiatives | 36.40 (4) | 25 (1) | 42.90 (3) | x^2^(1)=0.35, p=0.55 |
| Contact with ACT pre-admission, % *(n)*^a^ | 81.8 (9) | 75 (3) | 85.7 (6) | x^2^(1)=0.19, p=0.65 |

^a b^Data obtained from electronic health records. ^b^Calculated for participants presenting cirrhosis and decompensated liver disease ^c^Calculated for participants presenting decompensated liver disease

ACT: Alcohol Care Team; APQ: Alcohol Problems Questionnaire; ARLD: alcohol-related liver disease; AST: aspartate aminotransferase; CDAT: community drug and alcohol treatment; CM: contingency management; CPG score: Child-Pugh score; GGT: gamma-glutamyl transferase; INR: international normalised ratio; KCH: King’s College Hospital; MELD 3.0 score: Model for End-Stage Liver Disease 3.0 score; n: number; SADQ: Severity of Alcohol Dependence Questionnaire; SD: standard deviation; TLFB: timeline followback.

Independent samples t-test calculated for continuous outcomes and Chi-Square tests for categorical outcomes. Significance level p<0.05.
